# Chemokines and Chemokine Receptors in Multiple Sclerosis

**DOI:** 10.1155/2014/659206

**Published:** 2014-01-02

**Authors:** Wenjing Cheng, Guangjie Chen

**Affiliations:** Department of Immunology and Microbiology, Shanghai JiaoTong University School of Medicine, Shanghai Institute of Immunology, Shanghai 200025, China

## Abstract

Multiple sclerosis is an autoimmune disease with classical traits of demyelination, axonal damage, and neurodegeneration. The migration of autoimmune T cells and macrophages from blood to central nervous system as well as the destruction of blood brain barrier are thought to be the major processes in the development of this disease. Chemokines, which are small peptide mediators, can attract pathogenic cells to the sites of inflammation. Each helper T cell subset expresses different chemokine receptors so as to exert their different functions in the pathogenesis of MS. Recently published results have shown that the levels of some chemokines and chemokine receptors are increased in blood and cerebrospinal fluid of MS patients. This review describes the advanced researches on the role of chemokines and chemokine receptors in the development of MS and discusses the potential therapy of this disease targeting the chemokine network.

## 1. Introduction

Multiple sclerosis (MS), which was first described by Carswell, has been believed to be a chronic neuroinflammatory autoimmune disease with a still unknown etiology [1–3]. It is characterized by central nervous system (CNS) dysfunction, visual disorder, and motor deficits. MS is the most common cause of neurological disability in young adults [4]. Typically, the disease usually starts at the age of 20–40, being twice common in women as men [5]. Although the course of MS is variable, it is believed that there are different four patterns including relapsing-remitting multiple sclerosis (RRMS), primary progressive multiple sclerosis (PPMS), secondary progressive multiple sclerosis (SPMS), and primary-relapsing multiple sclerosis (PRMS) [6–8].

The pathogenesis of MS is still not well understood. As a multifactorial disease, MS is caused by some combination factors including viral infection, environmental factors, genetic predisposition, and autoimmune inflammation. Furthermore, recent data suggest that autoimmune inflammation plays a more important role in the development of this disease [9]. Although both the humoral and cellular immune responses are involved in the demyelinated tissue in MS, it is widely held that the cellular immune response is more crucial during MS development. Owing to the blood brain barrier (BBB) and blood cerebrospinal fluid barrier (BCB) involved, which provide an anatomic barrier to prevent free exchange of some substances between cerebrospinal fluid and blood to the CNS, the pathogenesis of CNS disease is different from other inflammatory diseases [5]. In some cases such as viral infection or inflammatory stimulation, the lymphocytes, which are mostly myelin-specific T cells, can migrate through the BBB to the brain and spinal cord after being activated in periphery. And then in the CNS, these pathogenic cells are reactivated and release abundant of proinflammatory cytokines, which can specifically interact with their receptors and cause axonal damaging and demyelination. Furthermore, more and more available data suggest that chemokines and chemokine receptors participate in the recruitment of macrophages and T lymphocytes into the CNS and it has been considered the most critical mechanism in the pathogenesis of MS [10–12]. Although the migration of pathogenic cells within the CNS parenchyma is still not clearly understood, this process may be directed by chemotactic gradients created by chemokines that diffuse from sites of production at foci of inflammation [13].

## 2. Chemokines and Chemokine Receptors

### 2.1. The Chemokine Family: Subgroups and Functions

Chemokines, also known as chemoattractant cytokines, are a large group of small basic proteins with the molecular weight between 8 and 14 kDa and characterized by attracting leukocytes into the sites of inflammation and infection [14]. Monocyte-derived neutrophil chemotactic factor (MDNCF), which is a potentially mediator of leukocyte-specific inflammatory response, was firstly found by Yoshimura and his colleagues in 1987 [15]. Since then, the chemokine family have been extensively studied and more than 50 different chemokines have been identified in humans [16, 17]. Based on the number and spacing of their cysteine residues involved in the formation of disulfide bonds, chemokines are divided into five groups including CC (*β*-chemokines), CXC (*α*-chemokines), XC (*δ*-chemokines, often called as C subfamily), CX3C (*γ*-chemokines), and CX chemokine [18]. Although the chemokines of CC, CXC, and CX3C family have four cysteines, XC chemokines only have two [19]. CC chemokines, which are the largest group containing two adjacent cysteine residues near their N-terminus, its genes are clustered on chromosome 17 in humans. In CX3C and CXC chemokine subfamily, there are one or three additional amino acids (represented 3X or X in their names) separating the first two of the four cysteine residues, and most of the CXC chemokines are clustered on chromosome 4 in human [20]. The fifth subfamily CX chemokine, which has recently been identified in zebrafish by Nomiyama in 2008, lacks one of the two N-terminal cysteine residues but retains the third and fourth [18].

Besides the genome or protein structure-based classifications, chemokines can be categorized into two major groups, the homeostatic and inflammatory chemokines according to the mode of expression and function [21]. Homeostatic chemokines are those who can be constitutively expressed at noninflamed sites and involved in relocation of lymphocytes in physiological conditions, while inflammatory chemokines are expressed by related cells in inflammatory conditions and mediate emigration of leukocytes to inflamed sites.

By using some new techniques, for example, gene knock-out, antibody blocking, and transgenic technology, many studies have demonstrated that chemokines are involved in many pathological and physiological processes, including T-cell differentiation and activation, cytokines secretion, tissue remodeling, tumor progression, and neural development [22–24]. Moreover, some independent researchers also found that many chemokines are associated with autoimmune diseases, including Graves' disease (GD), rheumatoid arthritis (RA), systemic lupus erythematosus (SLE), and MS [25, 26].

### 2.2. Chemokine Receptors: What Are They?

Chemokines exert their functions through the interaction with their receptors which belong to GTP-binding protein coupled receptor [27]. Each chemokine receptor has a 7-transmembrane structure and couples to G-protein for signal transduction within a cell. The first chemokine receptor was identified by Holmes et al. in 1991 and now nearly 22 different chemokine receptors have been discovered in human [28]. Chemokine receptor nomenclature follows that of chemokines, with chemokine receptors named CXCRn, CCRn, CX3CRn and XCRn for the ligands of CXC, CC, CX3C, and C families, respectively [29]. However, it is still confused whether there exists a specific subgroup of chemokine receptors for CX chemokines. Individual chemokine receptor can identify more than one chemokine ligand, and correspondingly, most of the chemokines can bind to more than one receptor, which forms a complex chemokine network in immune response [30]. Furthermore, five atypical chemokine receptors CCRL1, CCRL2, CXCR7, DARC, and CCBP2, initially known as “silent” or “decoy” receptors, have been identified in recent years. Due to their deficiency of signaling and functional activities, atypical chemokine receptors cannot evoke the cell migration directly. However, a recent study showed that these atypical chemokine receptors can shape the chemokine gradients via degradation, transcytosis, or local concentration of their cognate ligands and eventually induce leukocytes recruitment indirectly in tissues [31].

## 3. Chemokines and Chemokine Receptors Involved in MS

**Figure 1:**
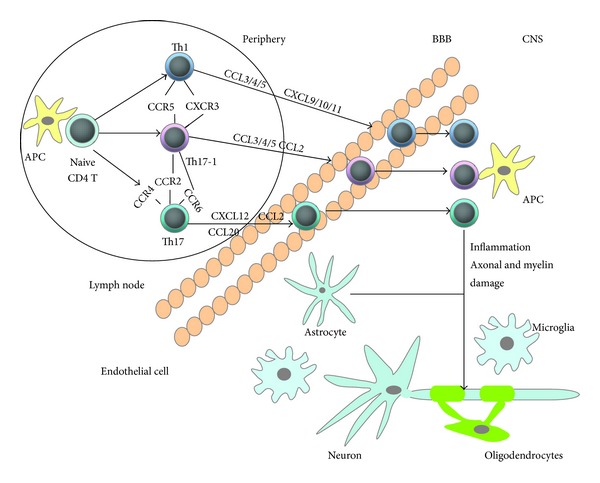
Migration and effector function of T cells in CNS during MS.

As a chronic autoimmune inflammatory disease, MS is specially characterized by demyelinating and neurodegeneration. A current consensus is that the infiltration, accumulation, and activation of myelin-specific T lymphocytes and macrophages in central nervous system are a vital aspect of MS pathology [32–34]. This inflammatory process is mainly mediated by CD4^+^ T cells, cytokines, chemokines, and chemokine receptors. Helper T cells can be divided into Th1, Th2, and Th17 subsets based on their characteristic cytokines-production patterns and effector functions. Th1 cells are responsible for cellular immunity and mainly release IFN-*γ* and TNF [35]. Th2 cells, which are often involved in humoral immunity, can produce cytokines such as IL-4, IL-5, and IL-10. Th17 cells mainly produce IL-17 and IL-6 and are responsible for inflammatory reaction. The chemokine receptor expression pattern would confer to each Th subset a unique characteristic of migration to corresponding ligand chemokines. Currently, a large number of researches have shown the immunoregulatory effect of chemokines and chemokine receptors on the development of MS [36–38] (Figure 1).  Table 1 lists the current considerable interesting chemokines and chemokine receptors involved in MS here.

We will focus on the Th1/Th2, Th17, and Th17-1 cells and related chemokines/chemokine receptors involved in MS as follow.

### 3.1. Th1/Th2 Cells and Related Chemokines/Chemokine Receptors Involved in MS

In 1989, CD4^+^ T cells were first divided into two subsets Th1 and Th2 [48]. Previous animal and human studies revealed that Th1 and Th2 lymphocytes and their related cytokines participate in the development of MS and EAE [49–51]. Th1 and Th2 cytokines can cross-inhibit each other and the progression of this disease may depend on the imbalance of Th1/Th2 ratio. It has been shown that in active phase of MS and EAE, Th1 cells can be found in lesions. As the level of Th1 cells is significantly increased in serum and CSF of MS patients, a shift from Th1 toward Th2 cytokine profile could have a beneficial effect on the clinical course of this disease [35]. Blockage of T-bet, which is the specific transcription factor of Th1 cells, will result in resistance to EAE in mice [39, 42].

During the last decade, a lot of studies detected the expression of chemokine receptors that are related to Th1 and Th2 cells as well as their relationship to MS and its animal model EAE [37, 52, 53]. It has been found that CCR5, CXCR3, and CXCR6 were preferentially, but not exclusively, expressed on Th1 cells, while CCR3, CCR4, CCR8, and CRTh2 (prostaglandin D_2_ receptor) were associated with Th2 cells [36, 37, 45, 54]. The levels of CXCR3 and CCR5 expressed on Th1 cells are increased in CSF and brain lesions of active demyelinating MS patients [41]. A potential reason is that the migration of T cells into the brain and spinal cord is meditated by the interactions between chemokine receptor and its ligand. Accordingly, CXCL10, which is the ligand of CXCR3 and expressed by astrocytes, can be detected in active lesions of MS [55]. Meanwhile the ligands of CCR5, CCL3, CCL4 and CCL5 are also detected in active MS lesions. The levels of CXCL10, CCL3, and CCL5 are considered to reflect the Th1 reactions. The changes of these chemokines expression in CSF are thought to represent the infiltration of Th1 cells [44]. Nakajima and collaborators have studied the expression of Th1/Th2-related chemokine receptors in MS patients and found that the ratio of CD4^+^CXCR3^+^/CD4^+^CCR4^+^,which represents Th1/Th2 balance, was higher in active MS patients than remission MS group, indicating that there is a shift from Th2 to Th1 in pathogenesis of MS [44]. And this result is consistent with the study conducted by Uzawa et al. in 2010 [41].

### 3.2. Th17 Cells and Related Chemokines/Chemokine Receptors Involved in MS

As a distinct novel T helper lineage, interleukin 17-producing effector T cells (Th17 cells), was found in 2005 [56, 57]. These cells can produce IL-17 and regulate inflammatory chemokine expression and response. Differentiation of naive CD4^+^ T cells to Th17 cells is driven by TGF-*β* and IL-6. STAT-3 is a necessary transcription factor to regulate the differentiation of Th17 and the expression of ROR*γ*t and ROR*α*, which are specific transcription factors of this lineage [58, 59]. In recent years, there are increasingly evidences to support that Th17 cells have an important role in autoimmune CNS inflammation and are involved in many inflammatory diseases such as MS and rheumatoid arthritis (RA) [42, 60]. The discovery of Th17 cells opens up new areas in autoimmunity research.

The present study showed that the number of Th17 cells is increased in CSF of RRMS patients in relapse phase compared with patients during remission. This result suggested that Th17 cells play a pathogenic role in the development of MS [60]. MS was regarded as a Th1-related disease before however, it should be regarded as Th1/Th17-mediated disease based on some novel findings [61, 62]. It has been generally accepted that chemokines and chemokine receptors, which usually expressed in pathogenic cells, are required for the migration of lymphocytes into the CNS [42]. Some studies showed that human Th17 cells are enriched in CCR4^+^CCR6^+^, CCR2^+^CCR5^−^, and CCR6^+^ populations [36, 63]. Yamazaki et al. recently found that the expression of CCR6 was regulated by TGF-*β*, ROR*γ*t, and ROR*α*. Th17 cells also express the CCR6 ligand CCL20, which is induced synergistically by TGF-*β* and IL-6, as well as requiring STAT3, ROR*γ*t, and IL-21 [64]. In human normal tissue, the researchers found that CCL20 is constitutively expressed by epithelial cells of choroid plexus, indicating that the recruitment of CCR6-expressed Th17 cells into CNS may interact with those constitutively expressed CCL20 in the early phase of the disease [42, 65]. In EAE, the expressions of CCR6 and CCL20 were upregulated in spinal cord during disease development. The number of infiltrating T cells in CNS significantly decreased in CCR6 knock-out EAE mice, suggesting that the CCR6-deficient autoreactive Th17 cells failed to migrate into the CNS [43, 64]. Th17 cells promote migration of Th17 and Treg in vitro in a CCR6-dependent manner by producing CCL20 [64]. However, there are also some contradictory findings in EAE. For instance, two studies described that the disease is milder EAE in CCR6^−/−^ mice than WT mice [42, 43], whereas in other groups, they found that CCR6^−/−^ mice developed severer EAE compared to control group [64, 66, 67]. The reasons for these contradictory findings are not well understood, and these may be caused by the different mouse strains or different methods they used to induce EAE.

### 3.3. Th17-1 Cells and Related Chemokines/Chemokine Receptors Involved in MS

Th17-1 cells, as a novel T-cell subset, can coexpress cytokines IFN-*γ* and IL-17. Human memory CD4^+^ lymphocytes have a tendency to expand into Th17-1 cells [68–70]. Dhodapkar et al. reported that dendritic cells (DC) were regarded as the most efficient inducers of human Th17-1 cells and this ability could be enhanced by the uptake of apoptotic tumor cells and some inflammatory cytokines such as IL-6, IL-1, and TNF [71, 72]. It was found that the lymphocytes have an increased propensity to expand into Th17-1 cells in the blood and brain tissue of relapsing MS patients. Th17-1 cells could preferentially cross the human BBB and accumulate in the CNS of mice during inflammatory events [70].

Just like other subsets, Th17-1 cells can also express chemokine receptors including CCR6, CCR2, and CXCR3 [45, 63]. Two studies reported that CCR2^+^CCR5^+^T cells, which were specifically involved in the development of MS but not in other noninflammatory neurologic diseases, produced a large quantity of IFN-*γ* and a small amount of IL-17, while CCR2^+^CCR5^−^ T cells produced a large quantity of IL-17 and a small amount of IFN-*γ* [36, 63]. In relapse phase of MS, the level of CCR2^+^CCR5^+^ Th17-1 cells is increased in CSF, due to that these cells have an ability to produce MMP-9 and OPN. CCR2^+^CCR5^+^ Th17-1 cells are more capable of invading the brain parenchyma than other T cells [36].

Activated Th1 cells and Th17 cells are thought to be the main culprit in MS. Th1 cells are IFN-*γ* producing and Th17 cells are IL-17 producing T lymphocytes. While Th17-1 cells are a novel T-cell subset producing both IFN-*γ* and IL-17. A large number of chemokines and chemokine receptors have been responsible for the migration of T cells in the development of MS. Th1 cells can express CXCR3 and CCR5, which is the receptor of chemokines such as CCL3/4/5 and CXCL9/10/11. Th17 cells can express chemokine receptors including CCR2/CCR4/CCR6, and Th17-1 cells express CCR2/CCR5/CCR6/CXCR3. The interactions between these chemokine receptors and their ligands could mediate effector T cells migrating into CNS. Then these effector T cells can produce inflammatory products and cytokines that damage the myelin and axons.

## 4. The Therapy Targeting Chemokines/Chemokine Receptors Involved in MS

Emerging evidences have demonstrated that the levels of chemokines and their receptors are increased in the brain tissue, blood and cerebrospinal fluid in different stages of MS patients [47]. The chemokines/chemokine receptors expressed in different subsets of Th cells have the ability of attracting inflammatory cells into CNS, which will result in severe nervous system dysfunction [46, 73]. Thus, chemokine network is becoming a potential target for effective treatment of MS.

In clinical studies, some drugs targeting related chemokines and chemokine receptors have showed effective treatment through regulation of the immune responses in MS patients [74–76]. First, methylprednisolone (MP), a glucocorticosteroid drug, plays an essential role in the treatment of MS patients. This effect is mainly due to its anti-inflammatory ability. MP can inhibit the activation of T cells, promote the apoptosis of immune cells, and decrease the migration of them into the CNS [77, 78]. Jalosinski et al. found that the migratory ability of CD4^+^CCR5^+^ T cells was impaired after treatment with MP in active phase of MS patients [79]. Diminished mean level of CXCR3 ligand CXCL10 was also observed in serum of MS patients after intravenous MP treatment [80]. Second, glatiramer acetate (GA), formerly known as copolymer 1, is widely used for treatment of MS via increasing the levels of Th2-related cytokines and CCR7 expression, decreasing Th1-related cytokines as well as the expression of CCR5, CXCR3, and CXCR6 on T cells [40, 76, 81–84]. Third, IFN-*β* has been proven as an effective drug for treatment of RRMS patients for many years [85–87]. Dhib-Jalbut et al. demonstrated that IFN-*β* treatment could reduce the expression of CCL5, CCL3, and CXCR3, which was associated with Th1 cells, increasing the expression of CCR4 which was often expressed by Th2 cells in MS patients [88, 89].

Although many effective drugs have been discovered to treat MS patients, it is still hard to find a single-agent and long-lasting effective drug to suppress this disease. The underlying reasons are that the complex chemokine network contains abundant ligands and receptors, and the chemokines often display multiple functions. More research should be done to investigate the accurate regulatory roles of some special chemokines and chemokine receptors involved in MS to find out more effective targets for treating MS.

## 5. Conclusion

The clinical courses and outcomes are distinctly diverse for treatment of MS. The migration of pathogenic cells such as T cells and macrophages to the site of lesions in CNS is a vital aspect in pathogenesis of MS. Chemokine, a small protein with chemoattractant property, may facilitate the infiltration of pathogenic cells into the brain and spinal cord. The pathogenesis of MS is becoming more and more unambiguous through studying these proteins, which could be new therapeutic targets for this disease. To obtain more effective treatment for the autoimmune disease, we need to identify the ideal therapeutic target or molecule which is solely expressed on some autoimmune effector cells. Only then, we will be able to balance therapeutic effectiveness against the immunosuppression which is untoward. Although the complexity of chemokine network is bewildering, we hope that further therapy of MS targeting chemokine network could open up new vistas.

## Figures and Tables

**Table 1:** Chemokines described in patients with MS.

Chemokine system name	Human ligand (old name)	Target cells	Chemokine receptors
CCL2 [33]	MCP-1	T cells, NK cells, B cells, monocytes, and dendritic cells	CCR1, CCR2
CCL3 [36]	MIP-1*α*	T and B cells, macrophage, and neutrophils	CCR1, CCR4, CCR5
CCL4 [39]	MIP-1*β*	T cells, microglia, and macrophage	CCR5
CCL5 [33]	RANTES	T cells, macrophage, eosinophils, and dendritic cells	CCR1, CCR3, CCR5
CCL7 [40]	MCP-3	T cells, B and NK cells, dendritic cells, and monocytes	CCR1, CCR2, CCR3, CCR5
CCL8 [40]	MCP-2	T cells, monocytes, and dendritic cells	CCR1, CCR2, CCR3, CCR5
CCL11 [17]	Eotaxin	T cells, dendritic cells, eosinophils, and basophils	CCR3
CCL17 [41]	TARC	T cells	CCR4
CCL19 [3]	ELC	T and B cells, and dendritic cells	CCR7
CCL20 [42]	LARC	T cells, monocytes	CCR6
CCL21 [43]	SLC	T and NK cells, dendritic cells	CCR7
CXCL1 [40]	GRO-*α*	monocytes, neutrophils	CXCR2
CXCL8 [41]	IL-8	monocytes, neutrophils, and fibroblasts	CXCR1, CXCR2
CXCL9 [44]	MIG	T and NK cells	CXCR3
CXCL10 [45]	IP-10	T and NK cells, macrophages, and astrocytes	CXCR3
CXCL11 [33]	I-TAC	T and NK cells	CXCR3, CXCR7
CXCL12 [46]	SDF-1	T and B cells, dendritic cells, and monocytes	CXCR4, CXCR7
CXCL13 [46]	BCA-1	T and B cells, dendritic cells, and monocytes	CXCR5
CX3CL1 [47]	Fractalkine	T and NK cells, monocytes	CX3CR1

## Abbreviations

BBB: Blood brain barrier
BCB: Blood cerebrospinal fluid barrier
CNS: Central nervous system
EAE: Experimental autoimmune encephalomyelitis
GA: Glatiramer acetate
GD: Graves' disease
GRTh2: Prostaglandin D_2_ receptor
IL: Interleukin
IFN-*β*: Interferon-*β*
MDNCF: Monocyte-derived neutrophil chemotactic factor
MS: Multiple sclerosis
MP: Methylprednisolone
MMP-9: Matrix metalloproteinase-9
RA: Rheumatoid arthritis
SLE: Systemic lupus erythematosus
TGF-*β*: Transforming growth factor-*β*
TNF: Tumor necrosis factor
ROR-*α*: Retinoic acid-related orphan receptor-*α*
ROR-*γ*t: Retinoic acid-related orphan receptor-*γ*t
OPN: Osteopontin
DC: Dendritic cells
TGF-*β*: Transforming growth factor-*β*
DARC: Duffy antigen receptor group.
